# Non-ionotropic signaling by the NMDA receptor: controversy and opportunity

**DOI:** 10.12688/f1000research.8366.1

**Published:** 2016-05-26

**Authors:** John A. Gray, Karen Zito, Johannes W. Hell

**Affiliations:** 1 Center for Neuroscience, University of California, Davis, CA, USA; 2 Department of Neurology, University of California, Davis, CA, USA; 3 Department of Neurobiology, Physiology & Behavior, University of California, Davis, CA, USA; 4 Department of Pharmacology, University of California, Davis, CA, USA

**Keywords:** NMDA, Non-ionotropic, signaling, LTD

## Abstract

Provocative emerging evidence suggests that the N-methyl-d-aspartate (NMDA) receptor can signal in the absence of ion flux through the receptor. This non-ionotropic signaling is thought to be due to agonist-induced conformational changes in the receptor, independently of channel opening. Non-ionotropic NMDA receptor signaling has been proposed to be sufficient to induce synaptic long-term depression (LTD), directly challenging the decades-old model that prolonged low-level calcium influx is required to induce LTD. Here, we briefly review these recent findings, focusing primarily on the potential role of non-ionotropic signaling in NMDA receptor-mediated LTD. Further reports concerning additional roles of non-ionotropic NMDA receptor signaling are also discussed. If validated, this new view of NMDA receptor-mediated signaling will usher in an exciting new era of exploring synapse function and dysfunction.

## Introduction

N-methyl- d-aspartate receptors (NMDARs) are glutamate-gated cation channels that play crucial roles in neurodevelopment and bidirectional synaptic plasticity. Most of the functions of the NMDAR have been attributed to the influx of calcium ions during channel opening ^1– 10^. However, a flurry of recent studies ^11– 18^ have provided more systematic support for earlier studies ^19– 22^ suggesting that agonist binding to NMDARs can transmit information to signaling molecules independently of Ca ^2+^ influx through the channel. If confirmed, these findings may lead to new pharmacological approaches to target specific synaptic signaling cascades in the numerous disorders attributed to synaptic dysfunction, including autism, schizophrenia, post-traumatic stress disorder, epilepsy, addiction, and Alzheimer’s disease.

Synaptic plasticity is a well-established cellular model for learning and memory and involves the persistent increase (long-term potentiation, or LTP) and weakening (long-term depression, or LTD) of synaptic strength in response to various patterns of activity. At most excitatory synapses in the brain, NMDAR activation is important for the induction of both LTP and LTD ^23– 26^. The widely accepted model for how activation of a single receptor can produce opposite changes in synaptic strength involves the amount and duration of Ca ^2+^ influx ^27– 30^. This model posits that brief periods of high-frequency synaptic activity lead to a large, rapid increase in intracellular Ca ^2+^ through the NMDAR that activates a series of biochemical steps, leading to LTP ^31^. Conversely, prolonged periods of low-frequency synaptic activity drive a modest increase in Ca ^2+^ through NMDARs that activates a different series of biochemical steps, leading to LTD ^24, 25, 32^.

For NMDAR-dependent LTD, the model that a modest increase in intracellular Ca ^2+^ is necessary for LTD induction has recently been challenged. Using systematic pharmacological approaches that block ion flux but spare glutamate binding to the NMDAR, Nabavi *et al.* ^15^ found that LTD could still be induced in an NMDAR-dependent manner. Here, we will review this provocative finding and follow-up studies, highlighting a direct refutation ^33^ and confirmation ^16^ of this result. Furthermore, we will discuss possible explanations for the disparate findings and additional reports of NMDAR-mediated signaling independent of channel opening. Given the importance of NMDARs in synaptic development and plasticity, these findings have the potential to be transformative but need further detailed and rigorous follow-up.

## Does NMDAR-dependent LTD involve non-ionotropic mechanisms?

**Figure 1.**
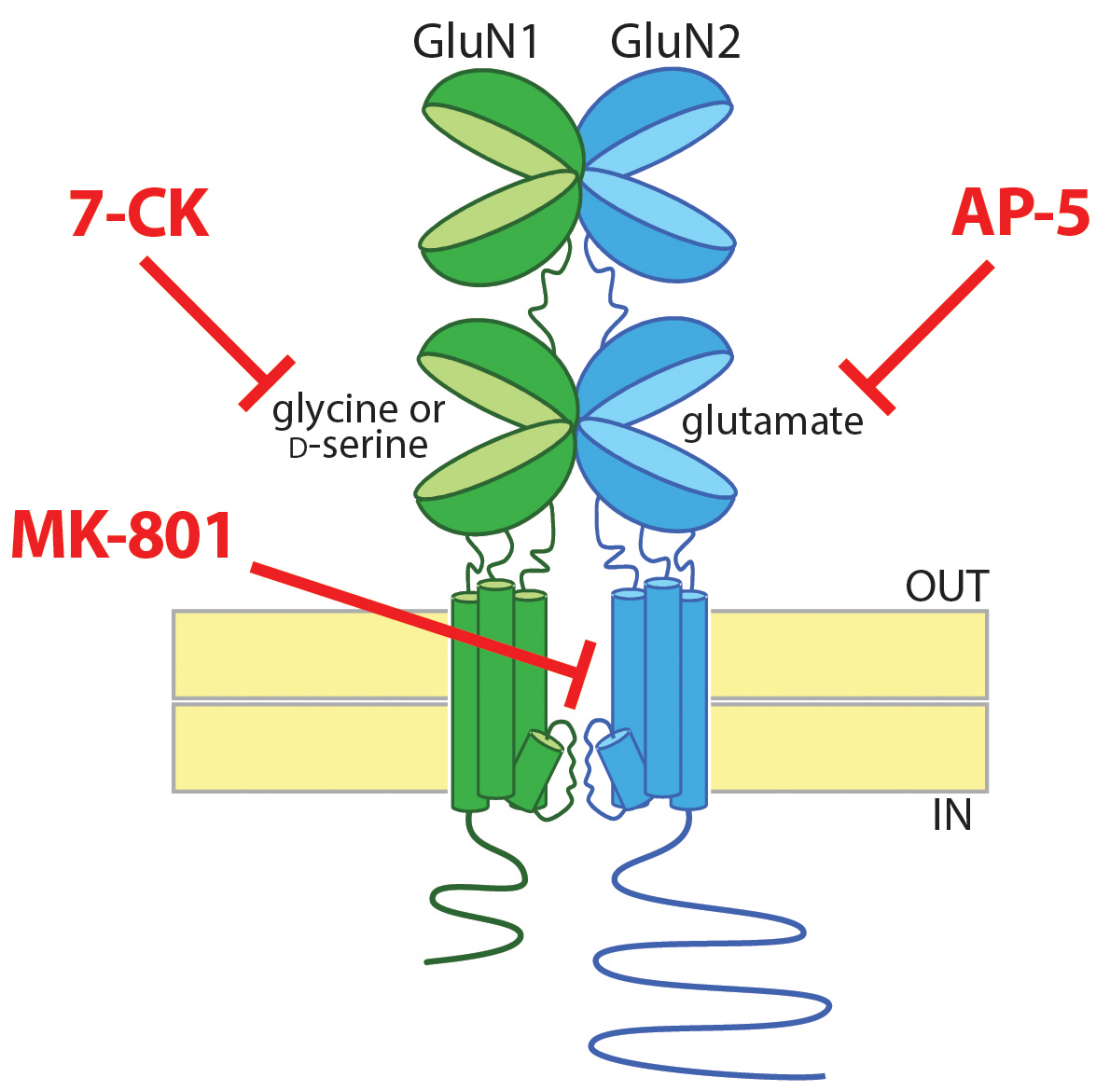
Pharmacology of the N-methyl- d-aspartate receptor (NMDAR). Most NMDARs are tetrameric proteins containing two GluN1 and two GluN2 subunits (for clarity, only one of each is pictured). For the NMDAR channel to open, both glutamate and a co-agonist, which can be glycine or d-serine, need to bind to clamshell-like ligand-binding domains on the GluN2 and GluN1 subunits, respectively. There are multiple approaches to block ion flow through the NMDAR channel: a competitive antagonist for the glutamate-binding site on the GluN2 subunits (e.g. AP-5), a competitive antagonist for the glycine/ d-serine binding site on the GluN1 subunits (e.g. 7-CK), or an uncompetitive blocker of the channel itself (e.g. MK-801). 7-CK, 7-chlorokynurenic acid; AP-5, (2R)-amino-5-phosphonovaleric acid; MK-801, dizocilpine.

NMDARs have a rich and complex pharmacology. Most NMDARs are heterotetramers containing two GluN1 subunits and two GluN2 subunits. One of the notable properties of NMDARs is that they are blocked by magnesium ions at resting membrane potentials ^35^; opening of NMDARs requires simultaneous activation by glutamate and depolarization to relieve the Mg ^2+^ block. In addition, NMDARs are unique among neurotransmitter receptors in having an absolute requirement for the binding of a co-agonist in addition to glutamate in order to open the channel. Glutamate binds to the extracellular ligand-binding domain on the GluN2 subunits, whereas the co-agonist, which is either glycine or d-serine, binds to the homologous ligand-binding domain on the GluN1 subunits ( Figure 1).

There are multiple approaches to blocking ion flow through the NMDAR: (1) a competitive antagonist of the glutamate-binding site on GluN2 (e.g. AP-5), (2) a competitive antagonist of the co-agonist site on GluN1 (e.g. 7-chlorokynurenate, or 7-CK), or (3) an uncompetitive blocker of the channel pore (e.g. MK-801) ( Figure 1). Using each of these pharmacologic strategies to block ion flow through the NMDAR, Nabavi *et al.* ^15^ surprisingly found that only the glutamate site antagonist AP-5 blocked LTD, suggesting that glutamate binding is required for LTD but not co-agonist binding or ion flow through the NMDAR. Although metabotropic glutamate receptor (mGluR)-mediated forms of LTD can also occur at these synapses ^36– 38^, application of an mGluR5 inhibitor and an inhibitor of L-type Ca ^2+^ channels (which are required for mGluR1-mediated LTD ^39^) did not block the LTD observed in the presence of 7-CK or MK-801, supporting the notion that signaling through mGluRs did not provide an alternate source for a rise in intracellular Ca ^2+^ leading to LTD.

So, what about the two decades of evidence that support a fundamental role for NMDAR-mediated Ca ^2+^ influx in LTD ^32^? Indeed, introducing Ca ^2+^ chelators intracellularly to the post-synaptic neuron prevents LTD ^24, 40– 43^. In addition, increases in intracellular Ca ^2+^ levels through activation of voltage-gated calcium channels ^44^ or photolysis of caged Ca ^2+^ ^30, 45^ induces synaptic depression that occludes additional LTD. Furthermore, LTD requires signaling mechanisms which are dependent on Ca ^2+^, including the activation of calcineurin ^46^ and hippocalcin ^47^. Nabavi *et al.* ^15^ also saw that Ca ^2+^ chelation inhibited LTD, but they proposed that the reduction in basal intracellular Ca ^2+^ concentration by strong chelation is responsible for the loss of LTD. To test this idea, they buffered the intracellular Ca ^2+^ concentration with the strong chelator BAPTA (1,2-bis(o-aminophenoxy)ethane-N,N,N′,N′-tetraacetic acid) along with additional free Ca ^2+^ in order to prevent acute rises in Ca ^2+^ while maintaining baseline Ca ^2+^ near physiological levels. This clamping of Ca ^2+^ at baseline levels did not block LTD, suggesting that low levels of basal Ca ^2+^, but not acute elevations of Ca ^2+^ through the NMDAR, are required for LTD. Remarkably, when Ca ^2+^ influx was blocked through the NMDAR with MK-801, a high-frequency stimulus that normally induces LTP actually resulted in LTD. Together, these results support that NMDAR-dependent LTD requires glutamate binding to the NMDAR but not Ca ^2+^ influx.

The surprising and provocative work by Nabavi *et al.* ^15^ was soon directly challenged. Specifically, Babiec *et al.* ^33^ found that, in contrast to Nabavi *et al.*, MK-801 effectively blocked LTD induction in slices from both young and adult animals. Furthermore, they showed that MK-801 blocked a chemical form of LTD induced by bath application of the glutamate site agonist NMDA. In addition, lowering extracellular Ca ^2+^ concentration also blocked LTD, supporting previous studies defining the role of Ca ^2+^ in LTD ^32^. These results stand in direct conflict with those of Nabavi *et al.*, and the reasons for these inconsistent findings remain unclear (see below for additional discussion).

Others have begun to weigh in. Recently, Stein *et al.* ^16^ examined the role of non-ionotropic NMDAR signaling in activity-induced dendritic spine shrinkage, a structural correlate of LTD. Using two-photon glutamate uncaging and time-lapse imaging, they found that low-frequency uncaging led to spine shrinkage even when the NMDAR glycine/ d-serine site was blocked with 7-CK. Furthermore, the presence of 7-CK or MK-801 converted high-frequency uncaging-induced spine enlargement to spine shrinkage, similar to the findings by Nabavi *et al.* for LTD. This spine shrinkage evoked in the presence of 7-CK was not inhibited by co-application of antagonists of mGluR1 and mGluR5. Importantly, since two-photon glutamate uncaging was used to bypass presynaptic neurotransmitter release, the potential effects of the pharmacological agents on pre-synaptic NMDARs were avoided. These results further support the idea that non-ionotropic NMDAR-mediated signaling mechanisms can drive synaptic depression.

Why the inconsistent results among laboratories ^48^? It is not yet clear. One possibility is that ionotropic and non-ionotropic mechanisms coexist. Alternatively, there may be unrecognized experimental differences (for example, in slice preparation, solutions, timing of drug application and removal, perfusion, and temperature). Indeed, a major limitation of these studies is the reliance on pharmacology. In most previous studies, AP-5 or other competitive glutamate site antagonists are used to block NMDAR activity, and we are unaware of any published examples in which AP-5 did not block NMDAR-mediated LTD. The examination of glycine co-agonist site antagonists in LTD, however, is a newer development, and it would have been enlightening if Babiec *et al.* had included one in their analysis. 7-CK is an early derivative of the naturally occurring kynurenic acid, which is a non-selective antagonist of all ionotropic glutamate receptors as well as the α7-nicotinic receptor. Although 7-CK is more potent and selective than kynurenic acid, it still exhibits a significant blockade of other receptors ^49^. Follow-up studies using higher-potency glycine site antagonists, and ones of different chemical classes, such as MDL 105,519 ^50^, will be of key importance.

Most of the conflicting results described above involve MK-801, an uncompetitive open-channel blocker of NMDARs ^51^. Oddly enough, it appears that this controversy is not new. Previous studies have shown an inhibition of LTD by MK-801 in the CA1 region of the hippocampus ^52, 53^ and the mouse visual cortex ^54^. However, an older report showed that although MK-801 blocked LTP, it did not block low-frequency stimulation-induced LTD in the hippocampus ^19^. Perhaps others have observed a lack of LTD inhibition by MK-801 but attributed this to experimental error or incomplete NMDAR blockade and therefore never reported it. Indeed, because of the use-dependent nature of MK-801, incomplete blockade of synaptic NMDARs may allow small local increases in Ca ^2+^ during repetitive stimulation right at the channel mouth that is not effectively buffered. Although this explanation is unlikely, it is difficult to rule out. Indeed, in an attempt to control for this use dependence of MK-801, slices are often incubated for a few hours in order to allow spontaneous activity to block all NMDARs prior to the onset of stimulation ^15, 33^, although others find that LTD is blocked when MK-801 is applied just minutes before induction ^55– 57^. Another issue is that the current tools for measuring changes in intracellular Ca ^2+^ are not sensitive enough to detect trace amounts of influx through the NMDAR, especially if that Ca ^2+^ is immediately bound to proteins within the NMDAR signaling complex. This could possibly be examined with a Ca ^2+^-impermeable or channel “dead” NMDAR or by attaching a genetically encoded Ca ^2+^ sensor directly to the NMDAR intracellular domains or associated proteins.

## Agonist-induced conformational changes in the NMDAR intracellular domains

Of course, the possibility of non-ionotropic signaling by NMDARs requires evidence of conformational changes upon agonist binding. While perhaps surprising for ligand-gated ion channels, non-ionotropic signaling is extremely common. G-protein-coupled receptors (GPCRs) comprise the largest protein superfamily in mammalian genomes and act solely through conformational changes upon extracellular agonist binding ^58, 59^. Indeed, the β2-adrenergic receptor, a prototypical GPCR, has only 168 intracellularly located amino acids, whereas NMDARs with their tetrameric structure and long complex C-terminal tails can have upwards of 1700 intracellular residues. In addition, at the post-synaptic density, NMDARs are a central member of a large macromolecular complex comprising signaling molecules, scaffolding and adaptor proteins, and cytoskeletal proteins ^60, 61^. Through these complex interactions, NMDARs are in a key position to engage and regulate intracellular signaling machinery. Indeed, while the long C-terminal tails of NMDARs have been presumed to be intrinsically unstructured, the complex scaffolding and interactions at the post-synaptic density may impart the secondary and tertiary structure ^62^ required to transmit information via agonist-induced conformational changes ^63^.

Recently, Dore *et al.* ^13^ demonstrated that NMDA binding to the glutamate site of the GluN2 subunits drives conformational changes in the NMDAR intracellular domains. Specifically, either green fluorescent protein (GFP) or mCherry was fused to the C-terminal tails of GluN1 subunits, and primary hippocampal neurons were co-transfected with both GFP- and mCherry-containing GluN1 subunits. Importantly, although the GluN2 subunits contain the glutamate-binding domain, GluN1 was chosen because tagging GluN2 subunits affects their trafficking and synaptic targeting ^64^. They then used fluorescence lifetime imaging microscopy (FLIM) to measure the lifetime of GFP fluorescence, which is reduced when in close proximity to mCherry because of Förster resonance energy transfer (FRET) ^65^. They found that NMDA caused rapid changes in GFP fluorescence lifetime in the presence of 7-CK or MK-801, but not in the presence of AP-5, providing evidence for agonist-induced, but ion flow-independent, conformational changes in the NMDAR C-terminal tails. In an accompanying study using similar techniques, Aow *et al.* ^11^ showed that NMDA binding, even in the presence of 7-CK (but not AP-5), leads to changes in the interactions between GluN1-GFP and various signaling proteins known to associate with the NMDAR which were tagged with the FRET acceptor mCherry. Specifically, they measured a rapid transient change in the interaction between GluN1 and protein phosphatase 1 (PP1) and a delayed but persistent change in the interaction of GluN1 with calcium/calmodulin-dependent protein kinase II (CaMKII). Together, these findings provide support that agonist binding to the NMDAR can produce intracellular conformational changes independently of ion flow through the receptor channel. These conformational changes are consistent with non-ionotropic signaling following glutamate binding to NMDARs.

## Other non-ionotropic NMDAR signaling

In addition to LTD, other studies have suggested additional roles for non-ionotropic NMDAR signaling. In Alzheimer’s disease, for example, impaired hippocampal synapse dysfunction is an early event ^66, 67^ that is associated with increased levels of diffusible oligomeric assemblies of the amyloid-beta (Aβ) protein ^68– 70^. These Aβ oligomers cause a rapid synaptic depression that is dependent on NMDAR activity ^71– 75^. Recently, it has been shown that this NMDAR-dependent, Aβ-induced synaptic depression does not require ion flux though the channel ^12, 14, 17^. Kessels *et al.* ^14^ found that increasing Aβ levels in organotypic hippocampal slice cultures through the viral expression of the β-secretase product of the amyloid precursor protein leads to a baseline synaptic depression that can be blocked by AP-5, but not 7-CK or MK-801. Similarly, Tamburri *et al.* ^17^ found that this synaptic depression can occur in acute hippocampal slices within 15 minutes of perfusion of oligomeric Aβ and that this rapid depression was dependent on NMDAR activity and was blocked by AP-5, but not MK-801, again suggesting that it did not require ion flux through the receptor ^17^. In addition to Aβ-induced synaptic depression, Aβ oligomer-induced synapse loss was recently demonstrated to be blocked by AP-5, but not MK-801 ^12^. Together, these results suggest that non-ionotropic NMDAR signaling contributes to the Aβ-induced synaptic dysfunction in Alzheimer’s disease and may suggest a common mechanism between Aβ-induced synaptic depression and non-ionotropic NMDAR-dependent LTD.

Other studies have described non-ionotropic NMDAR signaling, providing additional support for its physiological importance. In the earliest of these studies, glutamate binding was shown to induce dephosphorylation of the GluN2A subunit, resulting in the endocytosis of the receptor in the absence of ion flux ^21^. In another, co-activation of NMDARs and mGluR5 led to extracellular signal-regulated kinase (ERK) activation and increased c-Fos expression independent of ion flux but dependent on the interaction of the GluN2 C-terminal tail with scaffolding proteins in the post-synaptic density ^22^. NMDAR activation is also central to pathological processes that lead to neuronal death and non-ionotropic NMDAR-mediated signaling through Src kinase, and pannexin-1 was recently reported to occur during excitotoxicity ^18^. In addition to glutamate, co-agonist binding to the glycine site on the GluN1 subunits may also be involved in non-ionotropic signaling. For example, glycine or D-serine binding has been found to prime the NMDAR for subsequent clathrin-mediated endocytosis in the presence of AP-5 but not glycine site antagonists ^20^.

## A new horizon for NMDAR biology?

Here we have reviewed the current literature suggesting that activation of NMDARs can activate intracellular signaling independent of ion flux through the receptor. These results have been quite provocative, though from our perspective not entirely unexpected as NMDAR are part of a large multi-protein complex at the post-synaptic density and thus are in an ideal position to have conformation-based signaling. Although the physiological significance of potential parallel ionotropic and non-ionotropic NMDAR signaling processes remains controversial, their coexistence predicts the possibility for divergent signaling events based on agonist and co-agonist availability, channel opening, and receptor subunit composition. Ultimately, further exploration of this model may open a new frontier of NMDAR biology and lead to the development of novel approaches for targeting NMDAR signaling for the treatment of multiple neuropsychiatric disorders.

## Abbreviations

7-CK, 7-chlorokynurenic acid; Aβ, amyloid-beta; AP-5, (2R)-amino-5-phosphonovaleric acid; FRET, Förster resonance energy transfer; GFP, green fluorescent protein; GPCR, G-protein-coupled receptor; LTD, long-term depression; LTP, long-term potentiation; mGluR, metabotropic glutamate receptor; MK-801, dizocilpine; NMDA, N-methyl D-aspartate; NMDAR, N-methyl D-aspartate receptor.
